# *Actinotignum schaalii* Abscess in a Patient with Common Variable Immunodeficiency

**DOI:** 10.3390/pathogens9060494

**Published:** 2020-06-22

**Authors:** Christine Marie Panganiban, Sudhir Gupta

**Affiliations:** Division of Basic and Clinical Immunology, and Department of Pathology and Laboratory Medicine, University of California at Irvine, Irvine, CA 92660, USA; cmpanga@hs.uci.edu

**Keywords:** *Actinotignum schaalii*, common variable immunodeficiency, abscess, primary immunodeficiency

## Abstract

*Actinotignum schaalii* is an anaerobic, gram-positive commensal organism of the urogenital tract. *A. schaalii* typically causes urinary tract infections, predominantly in the elderly. Here, we describe the first case of *A. schaalii* infection presenting as cellulitis and abscess in a patient with common variable immunodeficiency. The patient was successfully treated with an incision and drainage and a prolonged antibiotic course. *A. schaalii* infection should be considered in sterile abscesses, and anaerobic cultures should be requested in the absence of positive routine cultures.

## 1. Introduction

In 1997, *Actinotignum schaalii* (formerly named *Actinobaculum* schaalii), a small facultative Gram-positive coccoid rod, was reclassified from the genus *Actinomyces* [1]. It is a relatively frequent commensal organism residing on the skin and mucosa of urogenital sites, but not in the colon [2]. *Actinotignum schaalli (A. schaalii)* is under-reported, as it is slow growing and difficult to phenotypically identify with typical microbiology laboratory techniques. Cultures should be incubated for 48–72 h on a blood agar plate in an anaerobic or a CO2-enriched environment [3]. It is increasingly being recognized as a cause of urinary tract infections in older adults [4], and rarely as a cause of abscesses [5]. 

Common variable immunodeficiency (CVID) is a heterogeneous primary immunodeficiency disorder characterized by low IgG and low IgA and/or IgM; impaired response to vaccines; and increased susceptibility to infections, enteropathy, autoimmunity, granuloma, and splenomegaly [6]. A number of gene mutations have been described; however, they account for only 15–20 of CVID patients [7,8]. Therefore, in the majority of cases, the genetic basis of CVID remains unknown. Among infections, upper and lower respiratory tract infections including pneumonia, chronic bronchitis, and sinusitis are the most common [9]. The most frequent organisms in CVID are *Streptococcus pneumoniae* or *Haemophilus influenzaa*, but *mycoplasma sp.* have also been noted in lungs, the urinary tract, and joints. Organ or tissue abscesses are rare in CVID, and no case of *A. schaalii* infection has been reported in CVID. Here, we describe the first case of an *A. schaalii* infection in a CVID patient presenting with cellulitis and an abscess of the abdomen. 

## 2. Case

A 47-year-old female, diagnosed with CVID according to the ESID/PAGID criteria [10], presented with a painful rash on her lower abdomen. She denied fevers, chills, and systemic signs of infection. On physical examination, the rash was erythematous and exquisitely tender, with a 10 mm × 5 mm area of induration with fluctuations. She was diagnosed with cellulitis and abscess. An incision and drainage was performed under sterile conditions. The abscess aspirate was transported anaerobically within an hour and sent for bacterial culture. The abscess aspirate was placed onto the following agars: sheep blood, chocolate, McConkey, and Columbia can, with colistin and nalidoxic acid and thioglyocolate broth. The plates were incubated in 5% CO2 at 35 °C. After 2 days incubation, growth on the sheep blood agar revealed small, white-grey, and smooth colonies. The organism was identified by matrix-assisted laser desorption ionization time-of-flight (MALDI-TOF) as *Actinotignum schaalii.* However, antibiotic sensitivity was not performed. After she was treated with sulfamethoxazole-trimethoprim (800–160 mg) for one tablet twice daily for 4 weeks, the cellulitis and abscess completely resolved. 

Previous medical history: Two years prior to the CVID diagnosis, she had seven episodes of urinary tract infections, six sinus infections, five episodes of bronchitis, five abscesses, and one episode of cellulitis. Urine samples from the seven urinary tract infection episodes were cultured using a standard microbiology culture method. All the samples grew *E. coli*. One of the prior episodes of abscesses was of right breast abscess. The abscess aspirate cultures grew few *Actinomyces* species; however, no further characterization of the species was performed. Since the urine and pus cultures were performed at some other facility, no detailed information about the culture methods was available. Associated morbidities included chronic obstructive pulmonary disease, type II diabetes mellitus, and hypothyroidism.

The subject gave her informed consent for inclusion before she participated in the study. The study was conducted in accordance with the Declaration of Helsinki, and the protocol was approved by the Ethics Committee (Institutional Review Board) of University of California, Irvine (HS-2001-2073).

Immunological data are shown in Table 1.

## 3. Discussion

Common variable immunodeficiency (CVID) is a heterogeneous primary immunodeficiency disease with increased susceptibility to infections, inflammation, autoimmunity, and malignancy [6,7]. A variety of microbial infections have been reported in CVID. However, an *A. schaalii* infection has not been reported in any of more than 423 different primary immunodeficiency diseases. Our patient is the first case of *A. schaalii* infection in CVID. 

*A. schaalii* is a slowly growing rod that is usually seen on gram stain, but cultures by standard microbiology laboratory methods are negative. Therefore, *A. schaalii* infections could be missed. In our patient, the gram stain was positive and cultures performed under anaerobic conditions grew *A. schaalii.*

*A. schaalii* typically causes urinary tract infections, especially in aged subjects [4]; however, acute pyelonephritis has been observed in children [11]. Pearce et al. [12] examined the urinary microbiome using 16S rRNA gene sequencing in women with and without urgency urinary incontinence (UUI). Several bacterial genera were more frequently sequenced and cultured from the urine of women with UUI, including *A. schaalii*. A number of infections with *A. schaalii* are polymicrobial [13,14,15].

*A. schaalii* presenting with urinary tract infections has also been reported in other immunocompromized states, including a patient with HIV infection with normal CD4 counts and an undetectable HIV viral load [16] and a patient with post renal transplant acute graft pyelonephritis [17]. Our patient also had recurrent urinary tract infections; all the cultures were positive for *E. coli*. However, the urine cultures may not have been performed under anaerobic conditions. Therefore, we cannot rule out the possibility of polymicrobial infection with *A. schaalii*. 

Abscesses with *A. schaalii* are relative rare and occur frequently in the groin region, possibly related to *A. schaalii* being a commensal organism in skin and mucosa of the urogenital tract [4]. However, *A. schaalii* abscesses may be more common than previously realized. Lotte et al. [18] reported *A. schaalii* in abscess pus from seven patients, four were monomicrobial, and three were polymicrobial. Prignet et al. [19] also reported one patient each with *A. schaalii* recurrent breast abscess, and with abdominal wall abscess. Beguelin and colleagues [20] reported skin abscesses in 10% of cases. *A. schaalii* abscess has also been reported in a patient with hidradenitis suppurativa [5].

Other clinical manifestations of *A. schaalii* infection include bacteremia, Fournier gangrene, infective endocarditis, native aortic endocarditis, vertebral osteomyelitis, and necrotizing fasciitis [13,21,22,23,24,25]. 

*A. schaalii* is typically susceptible to β-lactams and vancomycin and resistant to second-generation quinolones and sulfamethoxazole-trimethoprim [4]. However, Lotte et al. [15] reported that in 33% of samples, *A. schaalii* was sensitive to sulfamethoxazole-trimethoprim. This would be consistent with the complete response in our patients to four weeks of sulfamethoxazole-trimethoprim therapy by the resolution of cellulitis and abscess. 

Immune responses to *A. schaalii* are not known. Recurrent abscesses in our patient may suggest that antibodies play a role in defense against *A. schaalii* presenting with complications other than urinary tract infections, i.e., abscess. 

## 4. Conclusion

*A. schaalii* appears to be an emerging pathogen with clinical manifestations beyond urinary tract infections. In patients with an antibody deficiency and recurrent abscesses with abscess aspirates positive for gram stain but cultures negative by the standard microbiology culture method, a possibility of *A. schaalii* infection should be entertained and anaerobic extended culture or molecular methods should be performed to identify *A. schaalii*. A large cohort of patients with antibody deficiency and culture negative recurrent abscesses should be investigated for *A. schaalii* to determine if there is an increased susceptibility to *A. schaalii* infections.

## Figures and Tables

**Table 1:** Immunology features of the CVID patient.

Laboratory Test	Patient	Reference Ranges
Immunoglobulins (mg/dL)		
IgG	585 *	694–1618
IgM	36 *	65–298
IgA	277	81–350
IgG Subclasses (mg/dl)		
IgG1	312 *	342–1117
IgG2	185	147–524
IgG3	36	21–114
IgG4	16	6–88
Protective antibody titers (≥1.3 μg/mL) against *Streptococcus pneumoniae*	5/23	>20/23
Lymphocyte Subsets (/mm3)		
Lymphocytes %	31	14–44
Absolute lymphocytes	3441	900–3300
CD3+CD4+%	54	31–61
CD3+CD4+ number	1858	338–1,194
CD3+CD8+%	32	10–38
CD3+CD8+ number	1101	85–729
CD4+/CD8+ ratio	1.69	0.9–3.7
CD3%	86	62–84
CD3+ number	2959	619–1,847
CD19+ B%	8	5–26
CD19+ B number	275	51–473
CD56+ NK%	5	1–17
CD56+ NK number	172	12–349

* Low values.

## References

[1] Lawson P.A., Falsen E., Akervall E., Vandamme P., Collins M.D. (1997). Characterization of Some Actinomyces-Like Isolates from Human Clinical Specimens: Reclassification of Actinomyces suis (Soltys and Spratling) as Actinobaculum suis comb. nov. and Description of Actinobaculum schaalii sp. nov. Int. J. Syst. Bacteriol..

[2] Könönen E., Wade W.G. (2015). Actinomyces and Related Organisms in Human Infections. Clin. Microbiol. Rev..

[3] Bank S., Cattoir V., Lienhard R., Grisold A.J., Thomsen T.R., Reinhard M., Olsen A.B., Christensen J.J., Søby K.M., Prag J. (2014). Recommendations for optimal detection and identification of Actinobaculum schaalii in urine. APMIS.

[4] Lotte R., Lotte L., Ruimy R. (2016). Actinotignum schaalii (formerly Actinobaculum schaalii ): A newly recognized pathogen—review of the literature. Clin. Microbiol. Infect..

[5] Maraki S., Evangelou G., Stafylaki D., Scoulica E. (2017). Actinotignum schaalii subcutaneous abscesses in a patient with hidradenitis suppurativa: Case report and literature review. Anaerobe.

[6] Bonilla F.A., Barlan I., Chapel H., Costa-Carvalho B.T., Cunningham-Rundles C., De La Morena M.T., Espinosa-Rosales F.J., Hammarström L., Nonoyama S., Quinti I. (2015). International Consensus Document (ICON): Common Variable Immunodeficiency Disorders. J. Allergy Clin. Immunol. Pr..

[7] Aggarwal V., Banday A.Z., Jindal A.K., Das J., Singh S. (2019). Recent advances in elucidating the genetics of common variable immunodeficiency. Genes Dis..

[8] Lois A.G., Yel L., Cao J., Agarwal S., Gupta S. (2015). Common variable immunodeficiency associated with microdeletion of chromosome 1q41.1-41.3 and a deficiency of IP3 kinaseβ. Clin. Trans. Immunol..

[9] Oksenhendler E., Gérard L., Fieschi C., Malphettes M., Mouillot G., Jaussaud R., Viallard J., Gardembas M., Galicier L., Schleinitz N. (2008). Infections in 252 Patients with Common Variable Immunodeficiency. Clin. Infect. Dis..

[10] Conley M.E., Notarangelo L.D., Etzioni A. (1999). Diagnostic criteria for primary immunodeficiencies. Representing PAGID (Pan-American Group for Immunodeficiency) and ESID (European Society for Immunodeficiencies). Clin. Immunol..

[11] Andersen L.B., Bank S., Hertz B., Søby K.M., Prag J. (2012). Actinobaculum schaalii, a cause of urinary tract infections in children?. Acta Paediatr..

[12] Pearce M.M., Hilt E.E., Rosenfeld A.B., Zillox M.J., Thomas-White K., Fox C., Kliethermes S., Schreckenberger P.C., Brubaker L., Gai X. (2014). The female microbiome: Acomparision of women with and without urinary incontinence. MBio.

[13] Pedersen H., Senneby E., Rasmussen M. (2016). Clinical and microbiological features of Actinotignum bacteremia: A retrospective observational study of 57 cases. Eur. J. Clin. Microbiol. Infect. Dis..

[14] Thomas-White K.J., Gao X., Lin H., Fok C.S., Ghanayem K., Mueller E.R., Dong Q., Brubaker L., Wolfe A.J. (2018). Urinary microbes and post-operative urinary tract infection risk in urogynecological surgical patients. Int. Urogynecol. J..

[15] Tschudin-Sutter S., Frei R., Weisser M., Goldenberger D., Widmer A.F. (2011). Actinobaculum schaalii - invasive pathogen or innocent bystander? A retrospective observational study. BMC Infect. Dis..

[16] Vallet A., Noël N., Bahi R., Teicher E., Quertainmont Y., Delfraissy J.-F., Ferlicot S., Potron A., Goujard C., Lambotte O. (2017). Recurrent obstructive acute pyelonephritis: A rare form of Actinotignum (Actinobaculum) schaalii infection in a HIV-1 infected patient. Anaerobe.

[17] Gupta A., Gupta P., Khaira A. (2012). Actinobaculum schaalii pyelonephritis in a kidney allograft recipient. Iran. J. Kidney Dis..

[18] Lotte L., Lotte R., Durand M., Degand N., Ambrosetti D., Michiels J.-F., Amiel J., Cattoir V., Ruimy R. (2016). Infections related to Actinotignum schaalii (formerly Actinobaculum schaalii ): A 3-year prospective observational study on 50 cases. Clin. Microbiol. Infect..

[19] Prigent G., Perillaud C., Amara M., Coutard A., Blanc C., Pangon B. (2016). Actinobaculum schaalii: A truly emerging pathogen?: Actinobaculum schaalii: Un pathogène réellement émergent?. New Microbes New Infect..

[20] Beguelin C., Genné D., Varca A., Tritten M.-L., Siegrist H., Jaton K., Lienhard R. (2011). Actinobaculum schaalii: Clinical observation of 20 cases. Clin. Microbiol. Infect..

[21] Kus N.J., Kim B.J., Ross H. (2019). A case report of necrotizing fasciitis with growth of Actinomyces europaeus and Actinotignum schaalii. J. Surg. Case Rep..

[22] Loïez C., Pilato R., Mambie A., Hendricx S., Faure K., Wallet F. (2018). Native aortic endocarditis due to an unusual pathogen: Actinotignum schaalii. APMIS.

[23] Diallo K., Ferrand J., Goehringer F., Selton-Suty C., Folliguet T., Alauzet C., Lozniewski A. (2018). Closing the Brief Case: An Unusual Cause of Infective Endocarditis after a Urological Procedure. J. Clin. Microbiol..

[24] Haller P., Bruderer T., Schaeren S., Laifer G., Frei R., Battegay M., Fluckiger U., Bassetti S. (2007). Vertebral osteomyelitis caused by Actinobaculum schaalii: A difficult-to-diagnose and potentially invasive uropathogen. Eur. J. Clin. Microbiol. Infect. Dis..

[25] Vanden Bempt I., Van Trappen S., Cleenwerck I., De Vos P., Camps K., Celens A. (2011). Actinobaculum schaalii causing Fournier’s gangrene. J. Clin. Microbiol. Juin..

